# Controllable self-assembly of tyrosine-rich triblock peptides into robust collagen mimetic bioscaffolds for aging skin rejuvenation

**DOI:** 10.1093/rb/rbae085

**Published:** 2024-07-16

**Authors:** Linyan Yao, Biyang Ling, Wenjie Huang, Qi Wang, Xiangdong Cai, Jianxi Xiao

**Affiliations:** State Key Laboratory of Applied Organic Chemistry, College of Chemistry and Chemical Engineering, Lanzhou University, Lanzhou 730000, China; School of Life Science, Lanzhou University, Lanzhou 730000, China; State Key Laboratory of Applied Organic Chemistry, College of Chemistry and Chemical Engineering, Lanzhou University, Lanzhou 730000, China; State Key Laboratory of Applied Organic Chemistry, College of Chemistry and Chemical Engineering, Lanzhou University, Lanzhou 730000, China; State Key Laboratory of Applied Organic Chemistry, College of Chemistry and Chemical Engineering, Lanzhou University, Lanzhou 730000, China; State Key Laboratory of Applied Organic Chemistry, College of Chemistry and Chemical Engineering, Lanzhou University, Lanzhou 730000, China; School of Life Science, Lanzhou University, Lanzhou 730000, China; State Key Laboratory of Applied Organic Chemistry, College of Chemistry and Chemical Engineering, Lanzhou University, Lanzhou 730000, China

**Keywords:** collagen mimetic bioscaffolds, tyrosine-rich peptides, skin rejuvenation

## Abstract

Skin aging, a complex physiological process characterized by alterations in skin structure and function, seriously affects human life. Collagen holds considerable potential in aging skin treatment, while animal-derived collagen poses risks of pathogen transmission. Self-assembled peptides have garnered increasing attention in creating collagen mimetic materials; however, previous reported self-assembled peptides rely on vulnerable non-covalent interactions or lack the capability of controlling morphology and incorporating functional motifs, limiting their ability to mimic collagen structure and function. We have herein created a controllable tyrosine-rich triblock peptide system capable of self-assembling into robust collagen mimetic bioscaffolds for rejuvenating aging skin. Through ruthenium-mediated crosslinking, these peptides self-assemble into well-defined nanospheres or collagen-mimetic scaffolds, precisely regulated by the triple-helical structure and tyrosine distribution. The self-assembled collagen mimetic scaffolds exhibit outstanding resistances to various solvents and pH conditions. The integrin-binding motif has been incorporated into the triple helical block without disrupting their assembly, while endowing them with superior bioactivities, effectively promoting cell adhesion and proliferation. *In vivo* studies demonstrated their efficacy in treating photoaging skin by accelerating collagen regeneration and activating fibroblasts. The self-assembled tyrosine-rich triblock peptides represent a versatile system for creating robust collagen mimetic biomaterials, providing great potential in skin rejuvenation and tissue regeneration.

## Graphical Abstract

**Figure:**
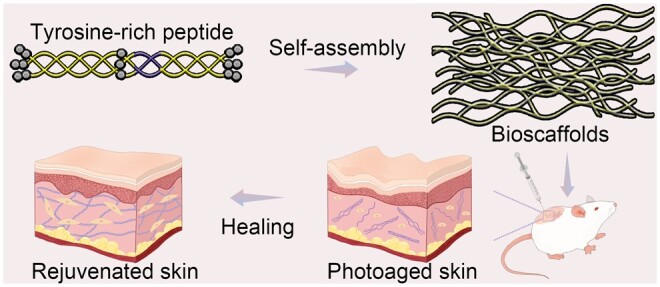

## Introduction

Skin aging represents a complex physiological process characterized by alterations in skin structure and function, profoundly influencing the human life [1, 2]. Various factors have been demonstrated to contribute to skin aging including natural aging, ultraviolet radiation and chronic diseases [3, 4]. Ultraviolet radiation has gradually emerged as the most prevalent cause of skin aging, owing to its ubiquitous presence, resulting in wrinkles formation, dermal laxity, inflammation and even skin cancer [5–7]. A few strategies have been attempted to improve the process of skin aging including topical skincare products, topical/oral antioxidant medications and laser therapy; however, they suffer from severe drawbacks, such as susceptibility to stability issues, limited efficacy in repair and a heightened risk of allergic reactions [8–13].

Collagen, as the most abundant structural protein of the dermis, holds considerable potential for healing photoaging skin owing to its ample sources, high biocompatibility and bioactivity. It has been revealed that collagen fibers undergo fracture and degradation in photoaged skin, highlighting the crucial necessity of stimulating collagen regeneration [14, 15]. Previous studies have demonstrated that collagen with cellular activity contribute to healing photoaging skin by stimulating the regeneration of dermal collagen [16, 17]. However, animal-derived collagen encounters disadvantages such as pathogen transmission, immunogenicity and batch-to-batch variability, posing thorny limitations to clinical applications [18]. Therefore, the construction of collagen mimetic biomaterials has received tremendous attention.

Self-assembled peptides have attracted increasing interests in the construction of collagen mimetic materials due to their high reproducibility, low immunogenicity and convenient modification [19]. Various non-covalent strategies have been established to drive peptide self-assembly such as hydrophobic interactions [20, 21], electrostatic interactions [22, 23] and metal–ligand interactions [24–26], while their intrinsic vulnerability to environmental changes presents significant obstacles for biomedical applications. A few covalent self-assembled peptides have been explored to develop stable fibrils and particles through the crosslink of cysteine [27, 28]. However, the lack of functional motifs and nearly uncontrollable morphology significantly limit their ability to mimic the functions of natural collagen. A versatile and robust design of self-assembled peptides that can mimic the collagen structure and function to treat photoaging skin remains a challenging target.

Herein, we have for the first time developed a series of tyrosine-rich triblock peptides that can controllably self-assemble into robust collagen mimetic bioscaffolds for photoaging skin rejuvenation. These triblock peptides, featuring a central triple helical block and two terminal tyrosine-rich blocks, have been shown to assemble into well-defined nanospheres or collagen-mimetic scaffolds, precisely regulated by both the triple-helical structure and tyrosine distribution. The self-assembled collagen mimetic scaffolds demonstrate exceptional robustness to diverse solvents and pH conditions. Functional sequences such as integrin-binding motif GFOGER have been incorporated into the triple helical block without disrupting their assembly, while endowing them with superior bioactivities, effectively promoting the cell adhesion and proliferation. *In vivo* studies further demonstrated their efficacy in treating photoaging skin by reducing inflammatory response and accelerating collagen regeneration. The self-assembled tyrosine-rich peptides represent a robust strategy for creating stable collagen mimetic bioscaffolds, providing potential applications in skin rejuvenation and tissue regeneration.

## Materials and methods

### Peptide synthesis

Standard Fmoc solid phase synthesis method has been used to synthesize designed peptides. Briefly, during the synthesis process, a double coupling method, using 4 equivalents of Fmoc-amino acids, 6 equivalents of DIEA and 4 equivalents of activator reagents (HBTU and HOBt), has been employed to achieve the coupling of amino acids. Fmoc group was removed using 20% piperidine (DMF). Finally, the resin was incubated with 25% acetic anhydride (DMF) at room temperature for 25 min to achieve the N-terminal acetylated peptide. The resin was then treated with TFA/TIS/H_2_O (95:2.5:2.5) for 3 hr to remove the side-chain protecting groups and cleave the peptides from the resin. The peptides were precipitated using cold diethyl ether, then resuspended in cold diethyl ether and centrifuged to obtain the crude peptides. The crude products were purified using reverse-phase high-performance liquid chromatography on a C_18_ column, and their molecular weight was confirmed by MS (Table S1).

### Circular dichroism spectroscopy

Circular dichroism (CD) experiments were performed on a Chirascan Instrument (Applied Photophysics Ltd, England). Peptides of 300 μM were prepared in deionized water at pH 10.0. Wavelength scans were acquired from 190 to 260 nm with a 1 nm/step and an averaging time of 0.5 s. Thermal unfolding curves were recorded by monitoring the characteristic band at CD_225 nm_. The temperature was increased 1°C/step from 4°C to 60°C for tyrosine-rich triblock peptides (TTP) TTP1, TTP2, TTP3 and TTP4, or from 4°C to 80°C for TTP5, TTP6, TTP7, TTP8, TTP9, TTP10 and TTP11 at a heating rate of 10°C/hr. The first derivative of their thermal unfolding curves was defined as their melting temperature (*T*_m_).

### Scanning electron microscopy

Scanning electron microscopy (SEM) images of self-assembled tyrosine-rich triblock peptides were acquired using a Hitachi S-4800 scanning electron microscope (Hitachi Limited, Japan) with an operating voltage of 5.0 kV. Typically, 40 μl 3 mM Ru(bpy)_3_Cl_2_, 120 μl 30 mM ammonium persulfate and 40 μl of 1 mM peptide at pH 10 were mixed to prepare the samples. The mixture was exposed to visible light for 6 min. and then repeated centrifugation with water and ethanol. The resuspended samples were dried on a silica slice, and sputter-coated with AuPd for 2 min. The diameter of fibers and the nanospheres was measured using Image J software [29, 30].

### Differential scanning calorimetry

Five milligrams of self-assembled TTP11 assemblies were uniformly spread in a crucible, and protected with nitrogen. The temperature of differential scanning calorimetry (DSC) experiment was ramped up at a rate of 5°C/min within the range from 25°C to 200°C.

### Enzymatic digestion experiment

Ten milligrams of self-assembled TTP11 assemblies were treated with 5 U/ml type I collagenases for 1, 3, 7 and 14 days. At every time point, the assemblies were washed, centrifuged, lyophilized and re-weighed to determine the residual materials. The percentages were calculated to evaluate the durability of self-assembled TTP11 assemblies.

$$\text{MR}\%={\text{W}}_{\text{t}}/{\text{W}}_{0}\times \text{1}00\%.$$

Where MR%: material residual percentages; *W*_0_: initial weight; *W*_t_: weight of sample after enzymatic digestion for 1, 3, 7 and 14 days.

### Injectability tests

The injectability of self-assembled TTP11 assemblies was conducted by pushing force experiments. A concentration of 35 mg/mL self-assembled TTP11 assemblies was loaded into 1 ml sterile syringes, and then extruded by connected to the universal testing machine with a constant speed of 20 mm/min.

### *In vitro* cytotoxicity

A 100 μl of HeLa cell suspension was seeded into a 96-well culture plate with a density of 5 × 10^3^ cells/well and incubated to allow attachment. A 100 μl suspension of self-assembled TTP11 assemblies was prepared at five different concentrations (0, 0.05, 0.1, 0.15 and 0.2 mg/ml). A 100 μl of 10 mM PBS was used as control. The plate was then incubated for 24 hr (37°C, 5% CO_2_). A 100 μl of 10% CCK-8 was added and incubated for 20 min (37°C, 5% CO_2_). Tecan Infinite F200/M200 multifunction microplate reader (Tecan, Männedorf, Switzerland) was used to acquire the optical density at 450 nm. Cell viability was calculated as the ratio of the mean absorbance value of each condition to the mean absorbance value of the control group.

TTP11 assemblies were immersed in DMEM culture medium and incubated at 37°C for 24 hr to obtain TTP-11 extracts with the concentration of 35 mg/ml. A 100 μl of HHF-1 cell suspension was placed and incubated for 24 hr. A 100 μl of 10 mM PBS was used as control sample. After 24 hr, their cytotoxicity was tested using 100 μl 10% CCK-8.

### Cell adhesion

The bioactivities of self-assembled TTP11 assemblies were investigated by cell adhesion assay using HeLa cells and human fore-skin fibroblast cells (HFF-1). TTP11 assemblies, Type I collagen, and heat-denatured BSA were added to untreated 96-well plates for 24 hr to coat the wells. PBS buffer (10 mM, pH 7.4) was used to wash the wells for three times. A 100 μl of HeLa cells or HFF-1 cells in serum-free DMEM was added and incubated for 6 hr, respectively. After incubation, the wells were washed with 10 mM PBS to remove unattached cells. The number of adhered cells was measured by total DNA quantification assay (Hoechst 33258, Solarbio). Attached cells were lyzed though three freeze–thaw cycles using liquid nitrogen to fully release the DNA. Hoechst 33258 at a concentration of 5 μg/ml was added and incubated for 1 hr. The fluorescence was determined using a Tecan Infinite F200/M200 microplate reader (Tecan, Männedorf, Switzerland). The assays were performed in triplicate, and the data were expressed as the mean ± standard deviation. The test was conducted three times, and the data were expressed as mean ± standard deviation.

### Immunofluorescence staining

Fluorescence microscopy was further employed to examine the cell adhesion and spreading properties. Coverslips placed in the non-treated 24-well plate were coated with one thin layer of self-assembled TTP11 assemblies for at least 24 hr, and then washed with PBS for three times. HeLa cells or HFF-1 cells were seeded and incubated at 37°C for 4 hr, respectively. Cold 4.0% formaldehyde was used to fix adhered cells for 10 min. Permeabilize and block the cells with 0.1% Triton X-100 for 5 min, and 1% BSA in PBS buffer for 30 min, respectively. The cells were incubated with phalloidin-tetramethylrhodamine isothiocyanate (500 μl, 100 nM) for 1 hr and then Hoechst 33258 (500 μl, 5 μg/ml) for 10 min at 37°C, respectively. Images were acquired using a Leica DM4000B metallurgical upright microscope (Leica Microsystems Inc., Wetzlar, Germany).

### Construction of the photoaging skin model

All animal experiments were performed with protocols approved by the ethics committee of School of Life Science, Lanzhou University (No. EAF2022010). Thirty adult male Kunming mice weighing 30 ± 2 g were purchased from Animal Experimental Center of Lanzhou University. All animals were fed with standard laboratory diets. The mice were randomly divided into three groups: the blank group with no radiation and treatment, the UV group with UV radiation and without any treatment, and the TTP11 group with UV radiation and subcutaneous injection of self-assembled TTP11 assemblies. Samples for the TTP11 group were prepared with self-assembled TTP11 assemblies at a concentration of 35 mg/ml in the physiological buffer.

The photoaging skin model was established following the previous methods [31]. Briefly, the back fur of all mice was shaved and then depilated. The TTP11 group was subcutaneously injected with 100 μl of TTP11 assemblies. The UVA (40 W, 320– 400 nm) and UVB (40 W, 290–320 nm) lamps were utilized to irradiate the mice back skin from a distance of 30 cm. The mice were irradiated for 15 min, every 2 days, lasted for 8 weeks. The cumulative irradiation dose of UVA and UVB was 18.81 and 2.06 J/cm^2^, respectively. At the 8th week, mice were anesthetized and euthanized. The skin samples were collected from the implanted sites.

### DermaLab Combo evaluation

Combo skin videoscope was used to gain optical images of the mice skin. The ultrasonic images of mice skin were measured using Combo ultrasound probe. The hydration and transepidermal water loss (TEWL) were quantitatively measured using Combo hydration probe.

### Histological analysis

The collected skin tissues were fixed in 4% paraformaldehyde in pH 7.4 10 mM PBS for 72 hr and then embedded in paraffin. The tissues were sectioned to a thickness of 5.0 µm and placed on poly-lysine-treated glass slides. After deparaffinization, the sections were stained using H&E or Masson's Trichrome staining kits, and imaged using an automated upright fluorescence microscope (Olympus Corporation, Olympus BX63).

### Quantitative analysis of SOD activity, MDA content and Hyp content

The superoxide dismutase (SOD) activity and malondialdehyde (MDA) content were determined using colorimetric assay kits. Skin tissues were added to the extraction solution at a ratio of 0.1 g/ml, and then homogenized under ice bath. Centrifuge the homogenate for 10 min and collect the supernatant. The SOD activity and the MDA content were measured using a UV-1750 spectrophotometer, respectively.

Chloramine-T colorimetric method was employed to quantify the concentration of hydroxyproline (Hyp) in the mice skin. Twenty milligrams of dried skin tissues were treated with 2 ml 6M HCl at 85°C for 12 hr. Adjust the pH of the solution to 7.0, and then diluted to constant volume. Chloramine T was added to the solution and incubated at room temperature for 20 min. The *p*-dimethylaminobenzaldehyde was added and incubated at 85°C for 10 min, then cooled to room temperature. The OD value at 561 nm was measured, and the content of Hyp were determined according to the standard curve.

### Statistical analysis

The data were presented as the mean ± standard deviation (SD) and statistically analyzed by Student’s t-test. Statistical significance was defined as no significance (NS) >0.05, *P < 0.05, **P < 0.01, ***P < 0.001 and ****P < 0.0001.

## Results and discussion

### Design of self-assembled tyrosine-rich triblock peptides

**Figure 1.**
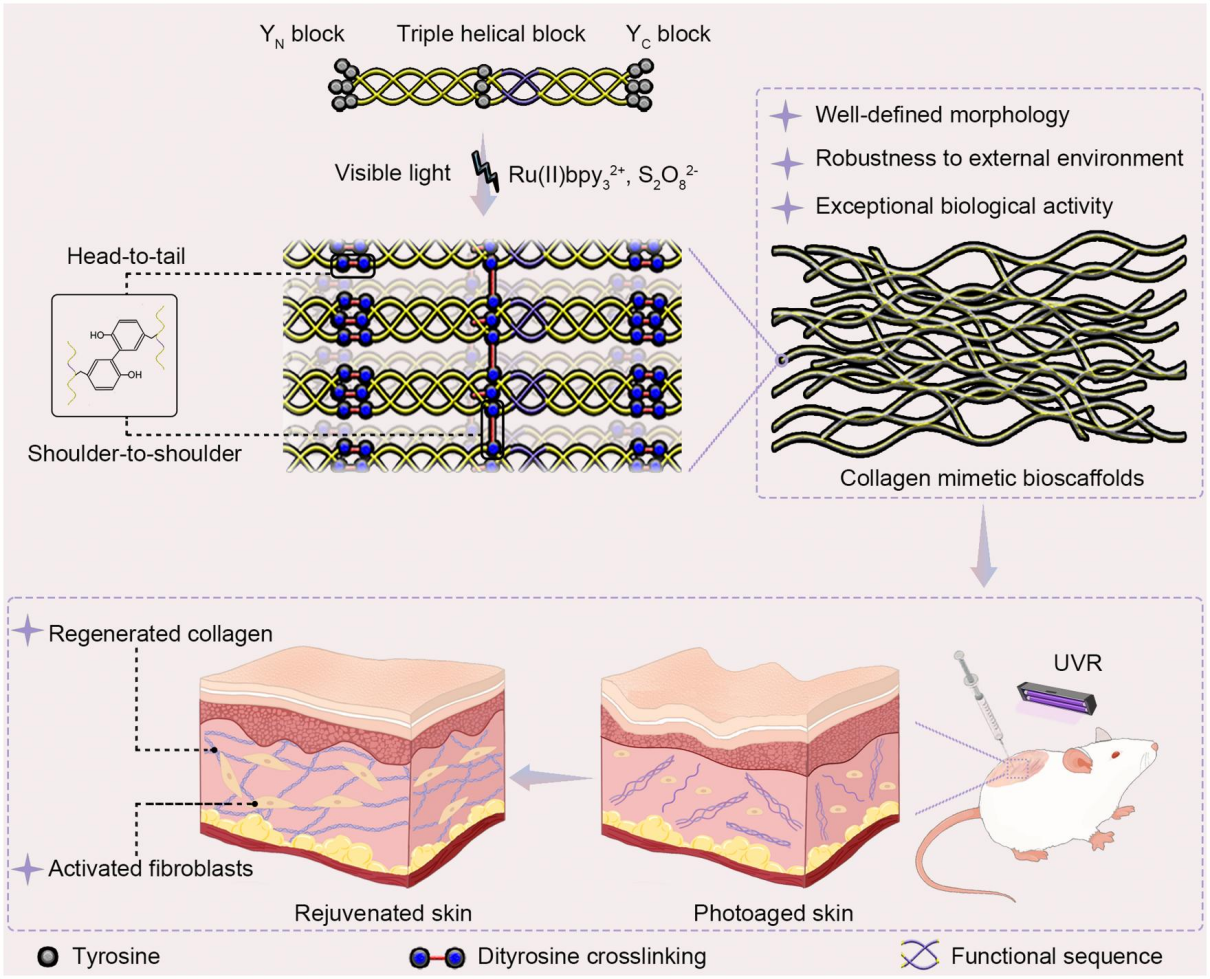
Schematic illustration of the self-assembly of tyrosine-rich triblock peptides into robust collagen mimetic bioscaffolds for photoaging skin rejuvenation.

A family of tyrosine-rich triblock peptides has been created to promote the self-assembly of peptides into robust collagen mimetic bioscaffolds for photoaging skin rejuvenation (Figure 1). The tyrosine-rich triblock peptides consist of three blocks: a central triple helical block containing at least one Tyr, and the N-/C-terminal blocks composed of Tyr, named as Y_N_ block and Y_C_ block, respectively. The self-assembly of the triblock peptides is initiated by the crosslinking of dityrosine, catalyzed by the Ruthenium tris-bipyridyl ions ([Ru(byp)_3_]^2+^) under visible light [32]. We hypothesize that the simultaneous incorporation of terminal and central tyrosine would promote the self-assembly of triple-helical triblock peptides into well-defined collagen mimetic scaffolds, while the inclusion of functional motifs would endow them with high bioactivity, effectively promoting the rejuvenation of photoaging skin (Figure 1).

Six tyrosine-rich triblock peptides Y_2_(GPO)_*m*_GYO(GPO)_*m*_Y_2_ (*m* = 0, 1, 2, 3, 4, 5; named as TTP1, TTP2, TTP3, TTP4, TTP5 and TTP6, respectively) have been designed to evaluate the effect of triple helical structure on their assembly (Table 1). Two peptides with zero and two tyrosines in the triple helical block (named as TTP7 and TTP8, respectively) and another two peptides without any terminal tyrosine (named as TTP9 and TTP10, respectively) have been constructed to examine the role of the central/terminal tyrosine in their assembly. Peptide TTP5-random, with the sequence YYGGOOPPOOPPGGGYOPPOOGGPPGGOOYY is designed to further study the influence of peptide length on the self-assembly (Table 1). The tyrosine-rich triblock peptides are postulated to be a highly versatile strategy to incorporate any functional motifs or desired amino acids in the triple helical block without meddling their self-assembly, as long as the tyrosine-rich pattern and triple helical structure is preserved. As a proof-of-concept demonstration, an example peptide TTP11 with the integrin-binding motif GFOGER has been created (Table 1). The biological function of the GFOGER motif requires the peptide to maintain a triple helix structure [33–36]. Therefore, we introduced GFOGER into the middle of the triple helical domain to ensure that it retains a stable triple helical conformation. To avoid the effect of crosslinking at the central Y on GFOGER function, we introduced a GPO sequence between the sequence GYO and GFOGER as a spacer.

**Table 1.** Design and thermal stability of tyrosine-rich triblock peptides

Name	Sequence	*T*_m_/°C
TTP1	YYGYOYY	<4
TTP2	YYGPOGYOGPOYY	<4
TTP3	YY(GPO)_2_GYO(GPO)_2_YY	<4
TTP4	YY(GPO)_3_GYO(GPO)_3_YY	25
TTP5	YY(GPO)_4_GYO(GPO)_4_YY	47
TTP6	YY(GPO)_5_GYO(GPO)_5_YY	57
TTP7	YY(GPO)_8_YY	50
TTP8	YY(GPO)_4_GYOGPOGYO(GPO)_4_YY	52
TTP9	(GPO)_4_GYO(GPO)_4_	51
TTP10	(GPO)_2_GYOGPOGYO(GPO)_3_	27
TTP11	YY(GPO)_4_GYOGPOGFOGER(GPO)_4_YY	28
TTP5-random	YYGGOOPPOOPPGGGYOPPOOGGPPGGOOYY	–

### Effect of triple helical structure on the self-assembly of tyrosine-rich triblock peptides

**Figure 2.**
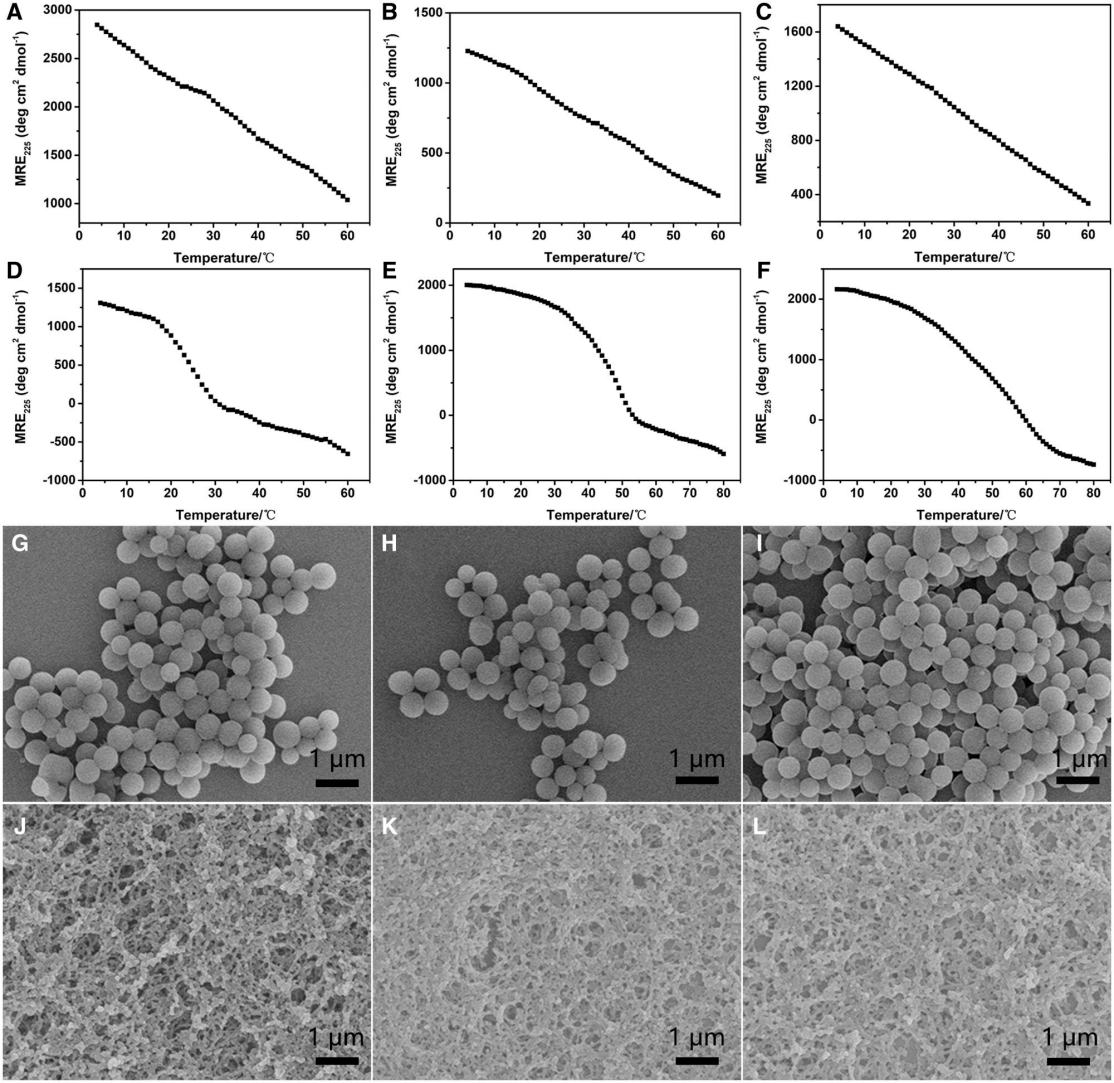
Effect of triple helical structure on the self-assembly of tyrosine-rich triblock peptides. CD thermal unfolding curves of peptides TTP1 (**A**), TTP2 (**B**), TTP3 (**C**), TTP4 (**D**), TTP5 (**E**) and TTP6 (**F**); SEM images of TTP1 (**G**), TTP2 (**H**), TTP3 (**I**), TTP4 (**J**), TTP5 (**K**) and TTP6 (**L**) assemblies.

CD spectra of peptides TTP1, TTP2, TTP3, TTP4, TTP5 and TTP6 with different lengths of Gly-Pro-Hyp triplets have been acquired to evaluate their triple helix and thermal stability (Figure 2A–F). Their thermal stability was determined by monitoring the ellipticity at 225 nm when gradually increasing the temperature. Peptides TTP1, TTP2 and TTP3 all displayed a linear thermal unfolding curve, suggesting that the three peptides maintained a monomer conformation (Figure 2A–C). In contrast, peptides TTP4, TTP5 and TTP6 displayed a distinct thermal transition, indicating the formation of stable triple helical structure with melting temperature (*T*_m_) values of 25°C, 47.0°C and 57.0°C, respectively (Figure 2D–F, Figure S1). These results demonstrated that the tyrosine-rich triblock peptides could form stable triple helix with a sufficient number of Gly-Pro-Hyp triplets (above 3) at both N-/C- terminals.

SEM was performed to study the morphology of self-assembled tyrosine-rich triblock peptides triggered by [Ru(byp)_3_]^2+^ (Figure 2G–I). A mixture of 0.2 mM peptide, 0.6 mM Ru(bpy)_3_Cl_2_ and 18 mM ammonium persulfate was prepared, and exposed to visible light for 6 min. All the peptide solutions initially appeared transparent, while a significant amount of aggregates were observed after visible light irradiation. SEM images indicated that peptides TTP1, TTP2 and TTP3 formed uniform nanospheres with diameters of 514 ± 45, 512 ± 50 and 509 ± 23 nm, respectively (Figure 2G–I). In contrast, SEM images of TTP4, TTP5 and TTP6 assemblies all displayed well-defined nanofibers with diameters of 52 ± 12, 55 ± 10 and 59 ± 10 nm, respectively (Figure 2J–L). These results suggested that the secondary conformation of the tyrosine-rich triblock peptides mediated the morphology of their assemblies, while triple helical structure is required for the formation of collagen mimetic scaffolds.

To further investigate the effect of triple helix on the self-assembly, peptide TTP5-random, containing a randomized Y_5_G_9_P_9_O_9_ sequence, was designed (Table 1). Initial transparent TTP5-random solution turned to form aggregates after the addition of [Ru(byp)_3_]^2+^ and the irradiation of visible light. SEM images of TTP5-random assemblies revealed the formation of nanospheres, indicating that a long peptide lacking of the triple helical conformation could not self-assemble into nanofibers (Figure S2). These results convincingly demonstrated that triple-helical conformation played a dominant role in mediating the morphology of peptide assemblies.

### Effect of tyrosine distribution on the self-assembly of tyrosine-rich triblock peptides

**Figure 3.**
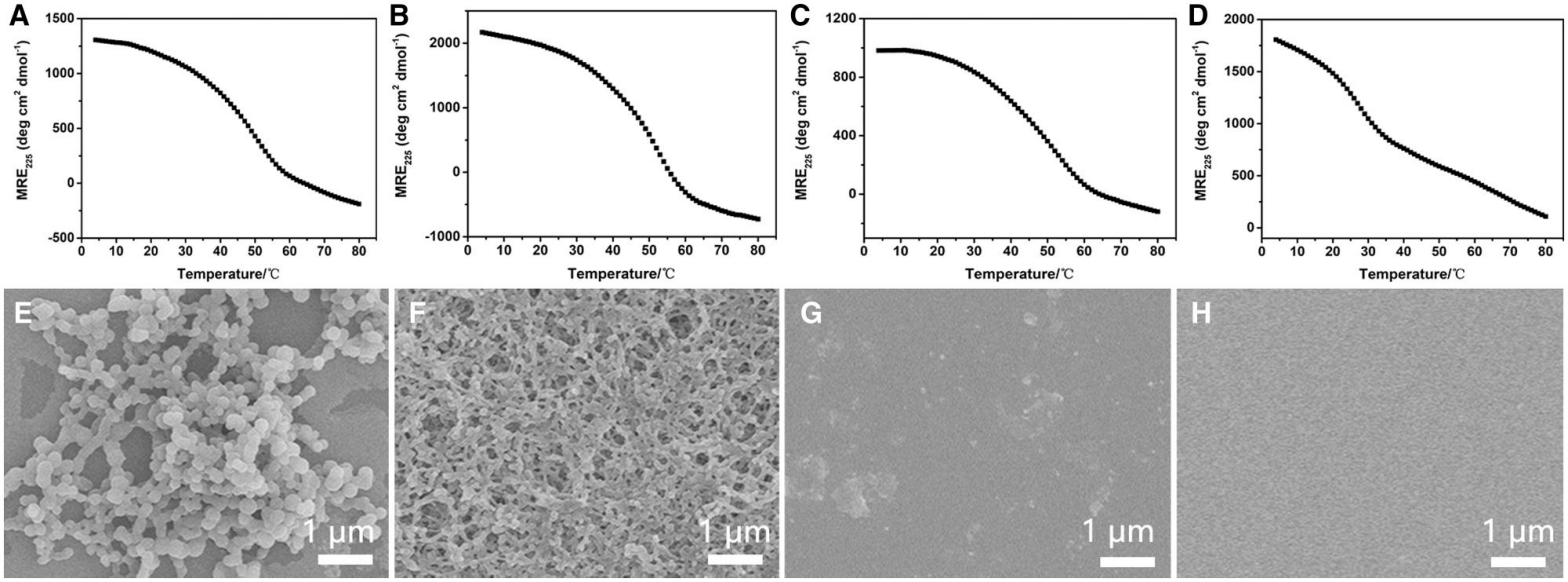
Effect of tyrosine distribution on the self-assembly of tyrosine-rich triblock peptides. CD thermal unfolding curves of peptides TTP7 (**A**), TTP8 (**B**), TTP9 (**C**) and TTP10 (**D**); SEM images of TTP7 (**E**), TTP8 (**F**), TTP9 (**G**) and TTP10 (**H**).

CD spectra of peptides TTP7, TTP8, TTP9 and TTP10 with different distribution of tyrosine have been carried out to examine their triple-helical stability (Figure 3A–D, Figure S3). All the four peptides displayed a characteristic CD band at 225 nm at 4°C. Thermal unfolding curves indicated the formation of stable triple helical structure with a *T*_m_ value of 50.0°C, 52.0°C, 51.0°C and 27.0°C, respectively (Figure 3A–D). After the addition of [Ru(byp)_3_]^2+^ and visible light irradiation for 6 min, the peptide mixtures of TTP7 and TTP8 turned cloudy, while TTP9 and TTP10 kept clear, demonstrating the terminal tyrosine played a determined role in the self-assembly of tyrosine-rich peptides.

The self-assembly of tyrosine-rich triblock peptides TTP7, TTP8, TTP9 and TTP10 was examined by SEM (Figure 3E–H). SEM images indicated that TTP7 formed nanospheres with a diameter of 201 ± 36 nm (Figure 3E), while TTP8 displayed well-ordered fibrous structures with a diameter of 58 ± 9 nm (Figure 3F), suggesting the tyrosine within the triple-helical block may not affect the capability of self-assembly, but it plays an important role in modulating the morphology of peptide assemblies. In contrast, SEM images of TTP9 and TTP10 indicated the absence of any significant nanostructures (Figure 3G and H), indicating that the terminal tyrosine is required to trigger the self-assembly of tyrosine-rich triblock peptide, while its absence would result in no aggregates. These results demonstrated that the head-to-tail and shoulder-to-shoulder assembly of tyrosine collectively determine the capability of triple-helical peptides to form collagen mimetic scaffolds.

### Robustness of self-assembled collagen mimetic scaffolds

**Figure 4.**
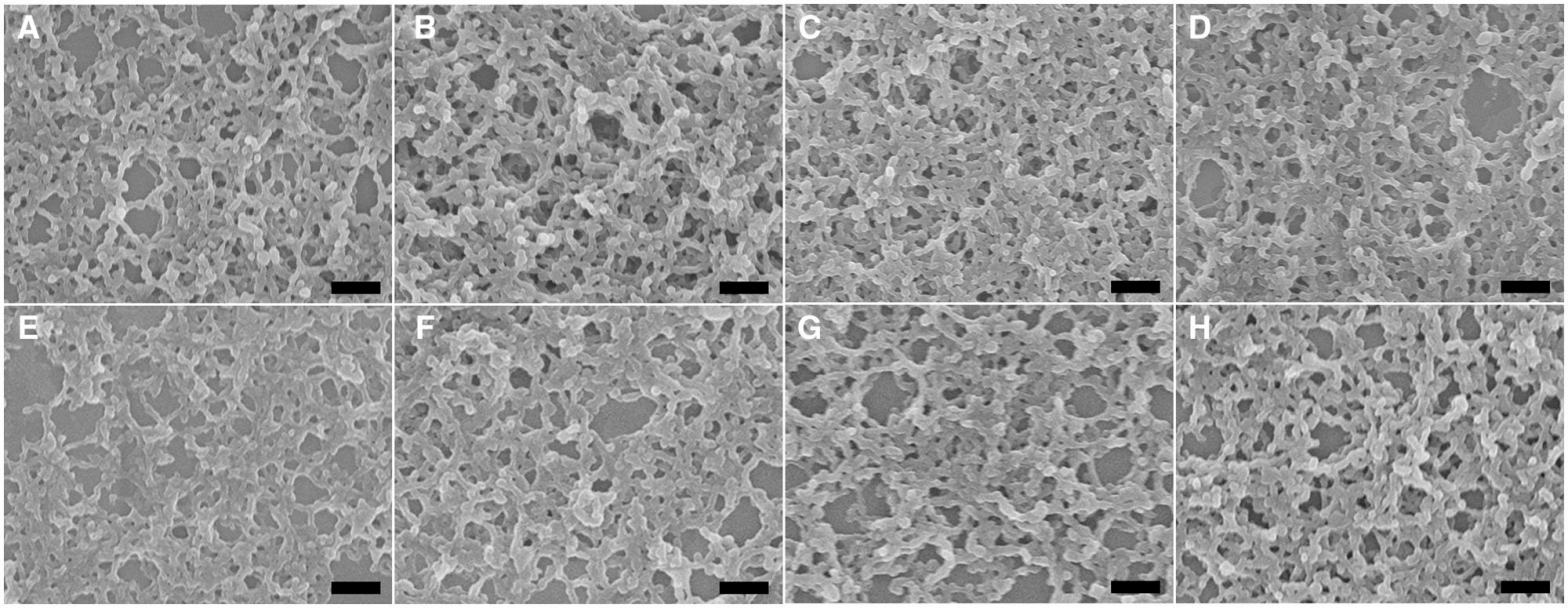
The robustness of self-assembled collagen mimetic scaffolds. SEM images of TTP5 assemblies incubated at different pHs: pH 4.0 (**A**), pH 6.0 (**B**), pH 8.0 (**C**) and pH 10.0 (**D**), and in different organic solvents: acetonitrile (**E**), tetrahydrofuran (**F**), dichloromethane (**G**) and xylene (**H**) for 24 hr at room temperature. Scale bar: 500 nm.

SEM images of self-assembled collagen mimetic scaffolds were examined under various pH and solvent conditions in order to evaluate their robustness to external environment (Figure 4). TTP5 was selected as a representative example of the triblock peptides, and its assemblies were subjected to different pH levels and organic solvents for 24 hr at room temperature. SEM images indicated that TTP5 assemblies maintained well-ordered fibrous scaffolds under broad pH conditions: pH 4.0 (Figure 4A), pH 6.0 (Figure 4B), pH 8.0 (Figure 4C) and pH 10.0 (Figure 4D). Furthermore, delicate fibrous scaffolds were well preserved in different organic solvents: acetonitrile (Figure 4E), tetrahydrofuran (Figure 4F), dichloromethane (Figure 4G) and xylene (Figure 4H). These results demonstrate that the self-assembly of tyrosine-rich triblock peptides provides highly robust collagen-mimicking scaffolds that are resistant to the external environment changes.

### Versatile incorporation of functional motifs into the self-assembled tyrosine-rich peptides

**Figure 5.**
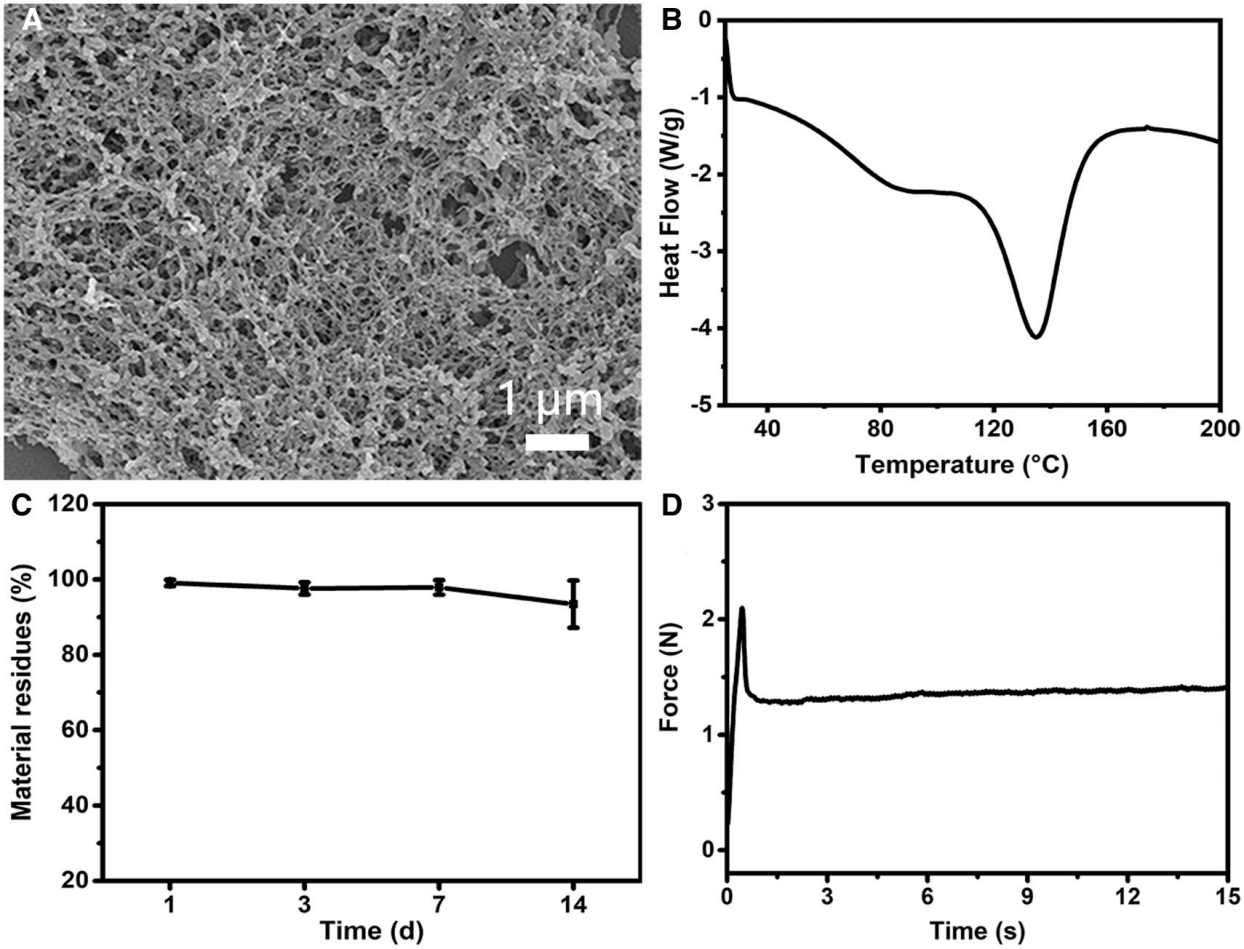
Characterization of self-assembled tyrosine-rich peptides with functional motifs. SEM image (**A**), thermal stability (**B**), durability (**C**) and injectability (**D**).

As a vivid example, peptide TTP11 has been created to investigate the versatility of tyrosine-rich triblock peptide system (Table 1). CD spectra was performed to evaluate the triple-helical stability of peptide TTP11. Its thermal unfolding studies displayed the formation of the stable triple helix with a *T*_m_ value of 28.0°C (Figure S4). Significant aggregates were observed after the addition of [Ru(byp)_3_]^2+^ and visible light irradiation. SEM images of self-assembled TTP11 assemblies indicated the formation of exquisite nanofibrous scaffolds (Figure 5A). The stability of TTP11 assemblies was further investigated by SEM after incubated under different pH for 24 h (Figure S5). SEM images indicated that TTP11 assemblies displayed delicate fibrous scaffolds in various pH conditions: pH 4.0 (Figure S5a), pH 6.0 (Figure S5b), pH 8.0 (Figure S5c), and pH 10.0 (Figure S5d). These results demonstrated the high versatility of the tyrosine-rich triblock peptides to conveniently incorporate biofunctional motifs or other desired amino acids without interfering in their assembly and stability.

The thermal stability of self-assembled TTP11 was investigated by DSC (Figure 5B). DSC curve displayed a thermal transition temperature of 134.6°C, indicating the self-assembly greatly enhanced their thermal stability. Enzymatic digestion assay was performed to examine the durability of the TTP11 assemblies (Figure 5C). The material residue percentages were monitored over a period of 1, 3, 7 and 14 days after treatment with type I collagenase. The TTP11 assemblies exhibited the residue percentage of 99.1%, 97.6%, 97.8% and 93.4% at day 1, 3, 7 and 14, respectively, demonstrating their remarkable durability (Figure 5C). Pushing force experiment, a key parameter for the injectability of implants, was conducted to evaluate the injectability of the TTP11 assemblies (Figure 5D). Previous study has reported that 10 N is the maximum acceptable injection force [37]. The TTP11 assemblies required an injection force of about 1.5 N at an extrusion speed of 20 mm/min, indicating their excellent injectability (Figure 5D). These results demonstrated that the TTP11 assemblies was a promising candidate for implant materials.

### *In vitro* cytocompatibility and cellular activity of self-assembled tyrosine-rich peptides

**Figure 6.**
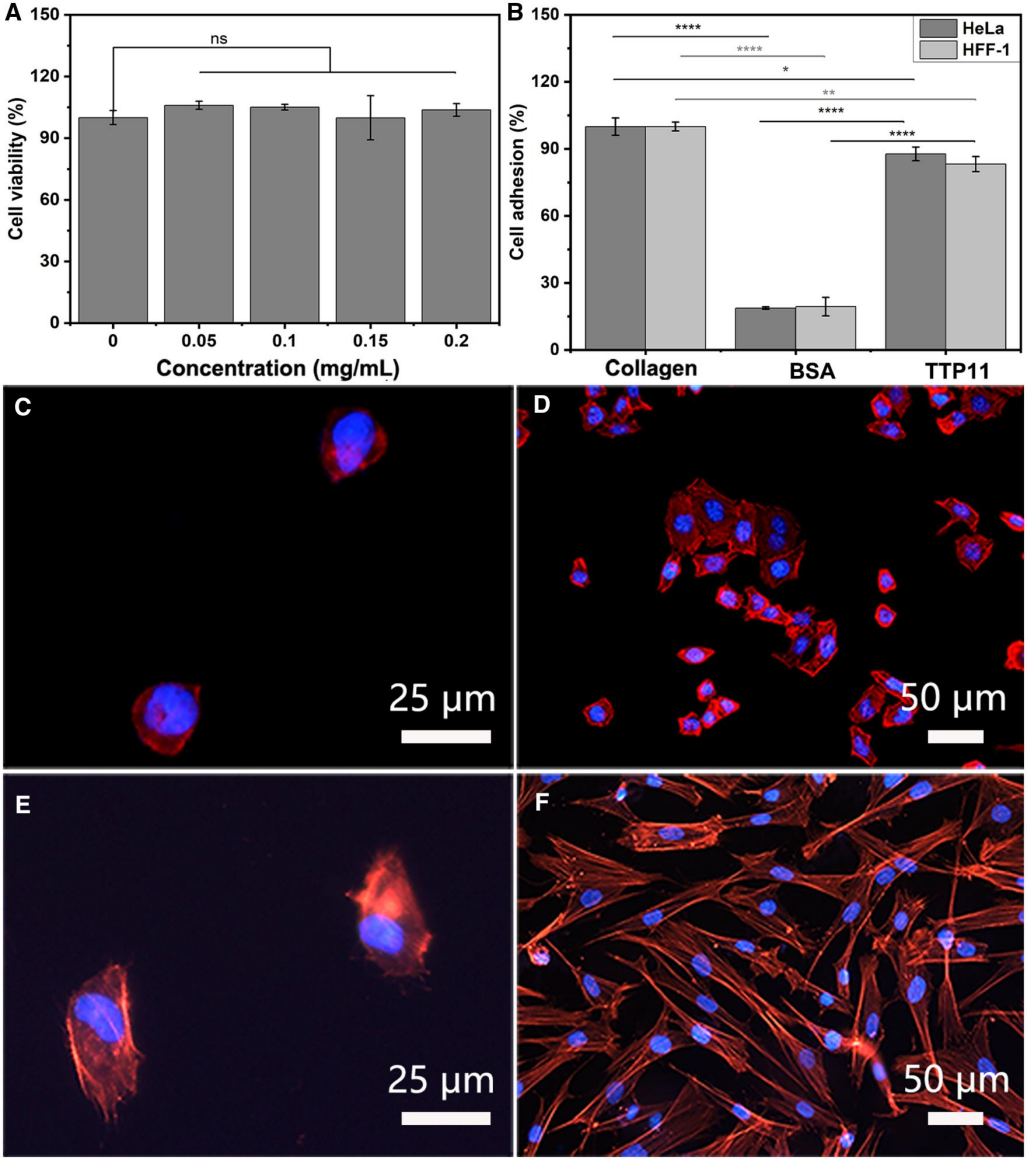
*In vitro* cytocompatibility and cellular activity of self-assembled tyrosine-rich peptides. Cytotoxicity of TTP11 assemblies (**A**); adhesion of HeLa cells (gray) and HFF-1 cells (light gray) on the surface coated with collagen, BSA and TTP11 assemblies (**B**); cell spreading of HeLa cells (**C**, **D**) and HFF-1 cells (**E**, **F**) on the surface coated with BSA (C, E) and TTP11 assemblies (D, F).

*In vitro* cytotoxicity of TTP11 assemblies was evaluated by detecting HeLa cells viability (Figure 6A). The TTP11 assemblies displayed similar cell viability at different concentrations from 0, 0.05, 0.10, 0.15–0.20 mg/ml, demonstrating their high cytocompatibility. The bioactivities of TTP11 assemblies were investigated by cell adhesion assay using HeLa cells and human foreskin fibroblast cells (HFF-1). Cells were cultured on the wells coated with type I collagen, heat-denatured BSA and TTP11 assemblies, respectively (Figure 6B). Adhesion to collagen substrate was taken as 100% reference level. The adhesion percentage of HeLa cells and HFF-1 cells to BSA was 18.7% and 19.4%, respectively, indicating that both types of cells barely bind to BSA. In contrast, the adhesion percentage of HeLa and HFF-1 cells to TTP11 assemblies were approximately 87.8% and 83.2%, respectively, demonstrating that the TTP11 assemblies possessed superior adhesion capability toward various types of cells.

The adhesion and spreading properties of the cells were further examined by fluorescence microscopy using HeLa cells and HFF-1 cells (Figure 6C–F). The attached cells were stained for actin stress fibers and nuclei with phalloidin tetramethyl rhodamine isothiocyanate and Hoechst 33258 nuclear dye, respectively. The fluorescence microscopy images indicated that the HeLa cells attached to BSA maintained the spherical shape, with less developed cytoskeletal structure (Figure 6C). In contrast, the HeLa cells on the TTP11 assemblies-coated substrates displayed well-developed actin cytoskeletal structure (Figure 6D). Similar phenomena were also observed for HFF-1 cells. The fluorescence microscopy images indicated that the HFF-1 cells on the BSA substrate displayed spherical shape with less developed cytoskeletal structure (Figure 6E), while the HFF-1 cells on TTP11 assemblies revealed a fine actin cytoskeletal structure (Figure 6F). The extensive cell adhesion and spreading suggested that the TTP11 bioscaffolds superbly recapitulate the cellular function of native collagen.

### Combo evaluation of the performance of self-assembled TTP11 bioscaffolds

**Figure 7.**
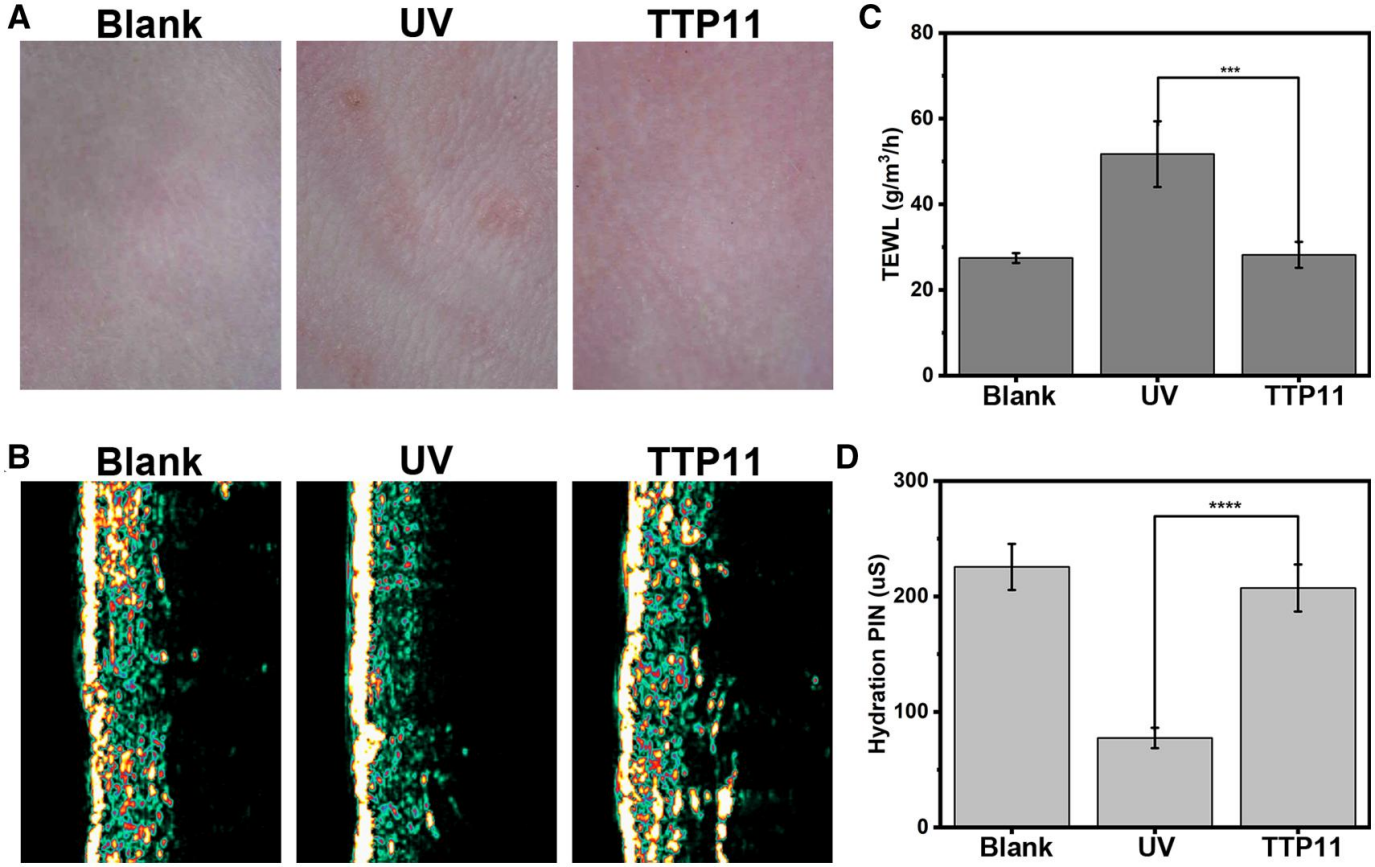
Combo evaluation of the performance of TTP11 assemblies on the skin rejuvenation. Representative skin microscopy (**A**), ultrasound skin imaging (**B**), TEWL values (**C**) and hydration values (**D**) of different mice groups: blank, UV and TTP11.

Thirty mice were randomly divided into blank group, UV group with UV radiation and without any treatment, as well as TTP11 group with UV radiation and injection of self-assembled TTP11 assemblies. The cytotoxicity of TTP11 assemblies was investigated by CCK-8 assay using their extracts with the concentration of 35 mg/ml (Figure S6). The TTP11 assemblies displayed similarly cell viability (97.37 ± 3.21%) with the blank group, indicating their high cytocompatibility. After 8 weeks, DermaLab combo was used to study the ability of TTP11 assemblies to promote photoaging skin rejuvenation. DermaLab Combo is a widely used skin testing equipment that integrates skin ultrasound, dermatoscopy and skin physiological indexes [38, 39]. Combo skin videoscope was conducted to investigate the appearance of mice skin (Figure 7A). The mice back skin of blank group showed no wrinkles; while the skin of UV group exhibited obvious wrinkles. The skin of TTP11 group appeared reduced wrinkles similar as the blank group. These results indicated that the self-assembled TTP11 bioscaffolds displayed remarkable durability and capability to fill the wrinkles.

Combo ultrasound probe was utilized to evaluate the ability of TTP11 scaffolds to improve the thickness and density of photoaging skin (Figure 7B). The color of Combo ultrasound images has been correlated with the density and thickness of skin in the order of white > yellow > red > blue > green > black [39, 40]. After 8 weeks, compared with the blank group, the skin of UV group exhibited broadened white region as well as diminished yellow and red regions, suggesting the increased epidermis thickness and decreased dermis density. In contrast, the skin of the TTP11 group appeared similar epidermis thickness and dermal density with blank group. These results indicated that self-assembled TTP11 bioscaffolds contributed to restoring the thickness and density of photoaging skin.

Combo hydration probe was used to evaluate the ability of TTP11 assemblies to promote the recovery of skin barrier function (Figure 7C). TEWL refers to the loss of water in the dermis through the epidermis, and the higher value indicated more water loss as well as worse skin barrier function [41, 42]. Compared to the blank group (27.5), the TEWL value of UV group significantly increased to 51.7, indicating the UV radiation greatly broke the skin barrier. In contrast, the TEWL value of TTP11 group significantly decreased to 28.2 (Figure 7C). The skin hydration of mice skin was further detected using the Combo hydration probe (Figure 7D). The higher the detected value indicated the higher water content in the stratum corneum. Compared to the blank group (225.5), the hydration value of UV group significantly decreased to 77.4, indicating the remarkable water loss. The hydration value of TTP11 group significantly increased to 207.2 (Figure 7D). These results convincingly indicated that the self-assembled collagen mimetic bioscaffolds could promote the barrier repair of photoaging skin.

### *In vivo* bioactivity of self-assembled TTP11 bioscaffolds

**Figure 8.**
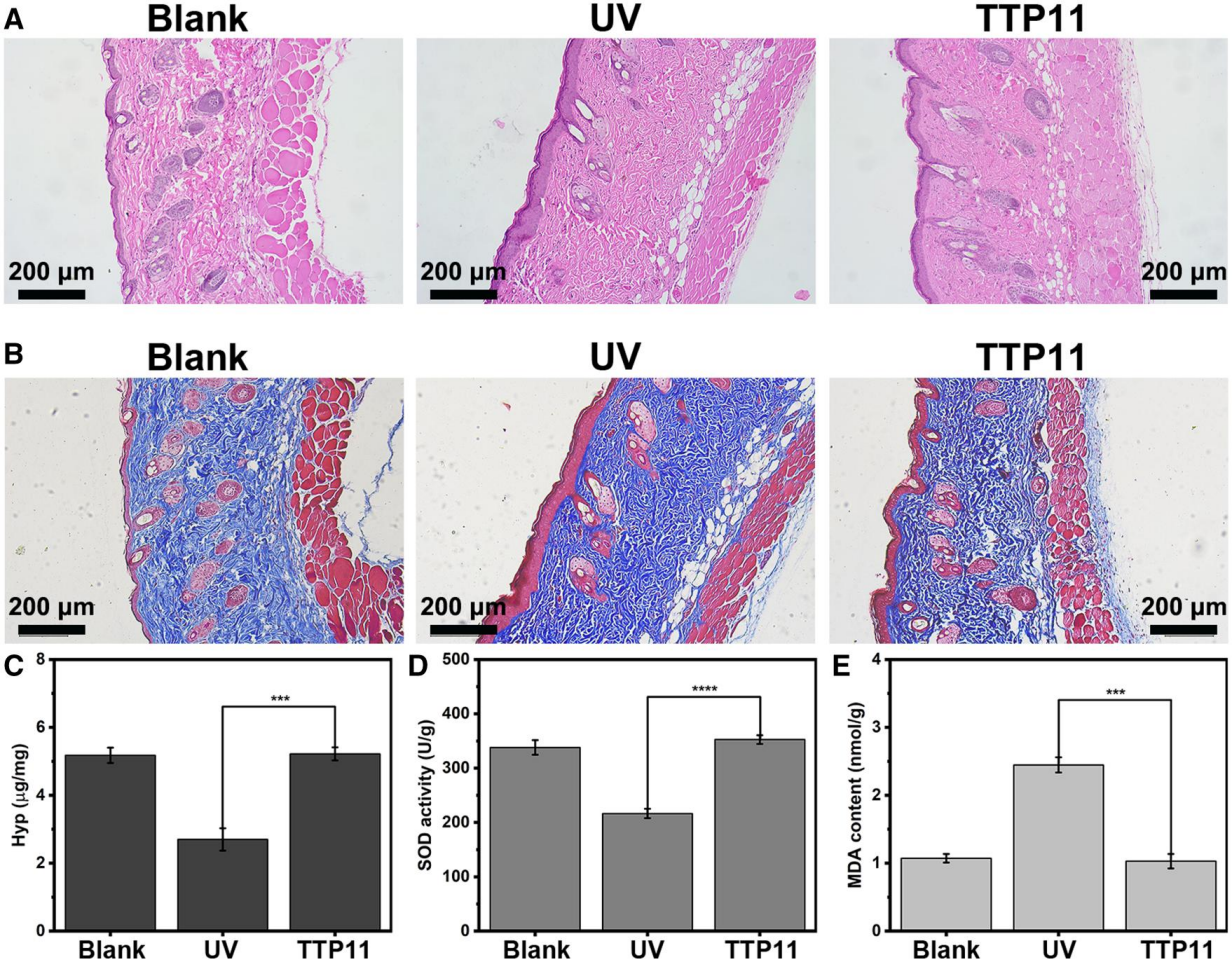
*In vivo* bioactivity of self-assembled TTP11 bioscaffolds. H&E staining (**A**), Masson staining (**B**), Hyp content (**C**), SOD activity (**D**) and MDA content (**E**) of blank, UV and TTP11 groups.

Histopathological analysis with H&E staining was conducted to assess the inflammatory response of the assembled TTP11, and its ability to promote the healing of photoaging skin (Figure 8A). The blank group mice showed no inflammatory reactions, and their back skin displayed a healthy condition with appropriate epidermal layer and orderly arranged cells, while the UV group exhibited abnormally thickened epidermis and remarkable inflammatory cell infiltration. In contrast, after 8 weeks of injections, the TTP11 group showed similar epidermal thickness as the blank group, and no infiltration of inflammatory cells. These results demonstrated that TTP11 assemblies resulted in remarkably reduced inflammatory response and excellent repair efficacies on the photoaging skin.

Masson's trichrome staining was employed to further investigate the ability of TTP11 assemblies to promote collagen regeneration (Figure 8B). The mice skin of blank group was observed in healthy condition with suitable epidermal layer and regularly arranged intact collagen fibers. The UV group displayed thickened epidermis as well as broken and disordered collagen fibers fragment. Conversely, the TTP11 group indicated the remarkable regeneration of collagen fibers. To further evaluate the capability of TTP11 assemblies to promoting the collagen regeneration, the hydroxyproline (Hyp) content was quantified (Figure 8C). After 8 weeks of injections, the Hyp content of blank group reached to 5.2 μg/mg, and the UV group showed a decreased value of 2.7 μg/mg. Remarkably, the TTP11 group exhibited significant increased value of 5.2 μg/mg. These results suggested that TTP11 assemblies possessed the ability to prevent the skin aging by accelerating the collagen regeneration and matrix remodeling.

The SOD activity and MDA content were measured to quantitatively study the ability of TTP11 assemblies to prevent the oxidative stress and promote the skin rejuvenation (Figure 8D and E). Previous study has reported that the excess UV exposure would result in the decrease of SOD activity and the increase of MDA content [43, 44]. Compared with the blank group (338.1), the SOD activity of UV group decreased to 216.5. In contrast, the SOD activity of TTP11 group was 352.6, even higher than the blank group (Figure 8D). Furthermore, the MDA content of UV group was increased to 2.5, while the TTP11 group showed the MDA value of 1.0, similar with that of blank group (1.1) (Figure 8E). These results convincingly revealed the exceptional efficacy of TTP11 assemblies on the photoaging skin rejuvenation by preventing the oxidative stress.

## Conclusion

We have herein for the first time developed a new family of self-assembled tyrosine-rich triblock peptides, which comprise three blocks including a middle triple helical block with at least one Tyr as well as the N-/C-terminal blocks composed of exclusively Tyr’s. The co-existence of the terminal and central Tyr’s enables the head-to-tail and shoulder-to-shoulder assembly of triple-helical triblock peptides, leading to the formation of collagen mimetic scaffolds. Compared to previously reported non-covalently assembled peptides, the covalent assembled peptides form extremely stable scaffolds that are highly resistant to diverse pH conditions and solvents, making them a well-suited biomaterial of clinical applications. Furthermore, the incorporation of the integrin-binding motif into tyrosine-rich triblock peptide endows their ability to mimic the structure, morphology, and function of collagen, greatly promoting the cell proliferation and adhesion.

Notably, the *in vivo* bioactivity of covalent assembled scaffolds has been investigated using photoaging skin mouse model. Combo evaluations indicate that TTP11 assemblies greatly promote the restoration of density, TEWL and hydration of photoaging skin to healthy levels. H&E and Masson staining images reveal that the self-assembled collagen mimetic bioscaffolds exhibited remarkable ability to prevent skin aging by reducing inflammation and stimulating collagen regeneration. The quantitative analysis of Hyp content, SOD activity and MDA content further demonstrated their bioactivity to accelerate the repair of photoaging skin. The self-assembled tyrosine-rich triblock peptides represent a versatile system to create robust collagen mimetic bioscaffolds, providing promising applications in the fields of skin rejuvenation and tissue regeneration.

## Supplementary Material

rbae085_Supplementary_Data
