# Supplementary material for: Controllable self-assembly of tyrosine-rich triblock peptides into robust collagen mimetic bioscaffolds for aging skin rejuvenation
Source: Regen Biomater. 2024 Jul 16;11:rbae085. doi: 10.1093/rb/rbae085 (PMC11333572; doi:10.1093/rb/rbae085)
Supplement: rbae085_Supplementary_Data [file rbae085_supplementary_data.docx]

Supporting Information

Controllable self-assembly of tyrosine-rich triblock peptides into robust collagen mimetic bioscaffolds for aging skin rejuvenation

Linyan Yao^a, b, 1^, Biyang Ling^a, 1^, Wenjie Huang^a^, Qi Wang^a^, Xiangdong Cai^a, b^, and Jianxi Xiao^a,^ *

^a^ State Key Laboratory of Applied Organic Chemistry, College of Chemistry and Chemical Engineering, Lanzhou University, Lanzhou 730000, China

^b^ School of Life Science, Lanzhou University, Lanzhou 730000, China

^1^ These authors contribute to the study equally.

^*^ Corresponding author. E-mail: [xiaojx@lzu.edu.cn](mailto:xiaojx@lzu.edu.cn)

Table S1. Mass spectroscopy characterization of tyrosine-rich triblock peptides.

| **Peptide name** |  | **m/z calculated** | **m/z found** |
| --- | --- | --- | --- |
| TTP1 | [M+Na]^+^ | 1068.0 | 1067.4 |
| TTP2 | [M+Na]^+^ | 1602.6 | 1602.1 |
| TTP3 | [M+Na]^+^ | 2137.2 | 2137.7 |
| TTP4 | [M+Na]^+^ | 2671.8 | 2671.0 |
| TTP5 | [M+Na]^+^ | 3205.3 | 3206.0 |
| TTP6 | [M+Na]^+^ | 3740.9 | 3741.0 |
| TTP7 | [M+Na]^+^ | 2873.0 | 2873.4 |
| TTP8 | [M+Na]^+^ | 3807.0 | 3807.1 |
| TTP9 | [M+Na]^+^ | 2553.7 | 2554.0 |
| TTP10 | [M+Na]^+^ | 2329.4 | 2351.8 |
| TTP11 | [M+H]^+^ | 4111.3 | 4111.1 |
| TTP5-Random | [M+Na]^+^ | 3205.3 | 3204.8 |


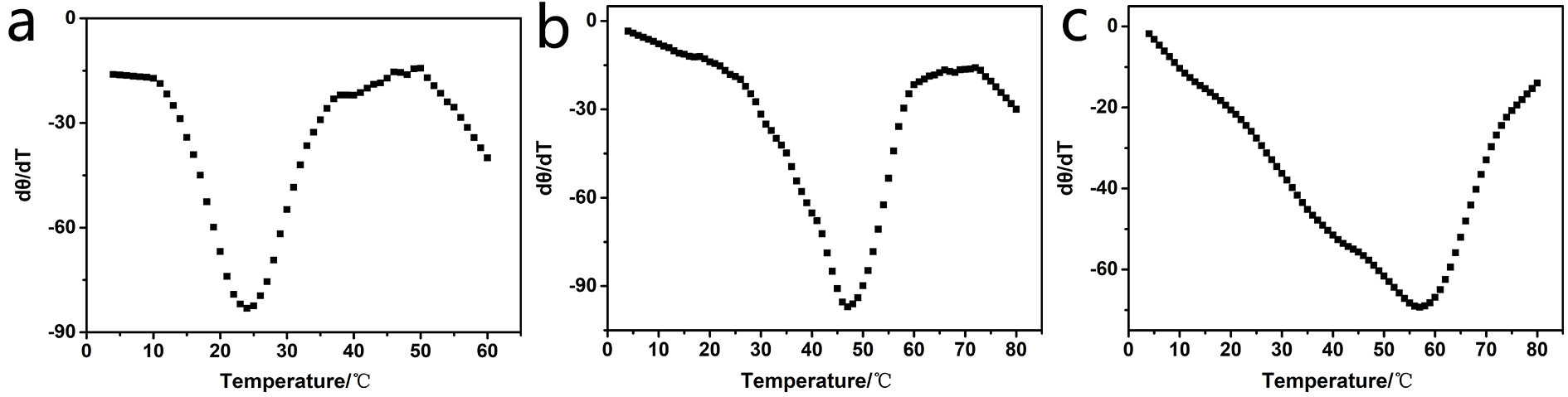


Figure S1. The first derivative (d[θ]/dT) of the CD unfolding curves of peptides TTP4 (a), TTP5 (b) and TTP6 (c).


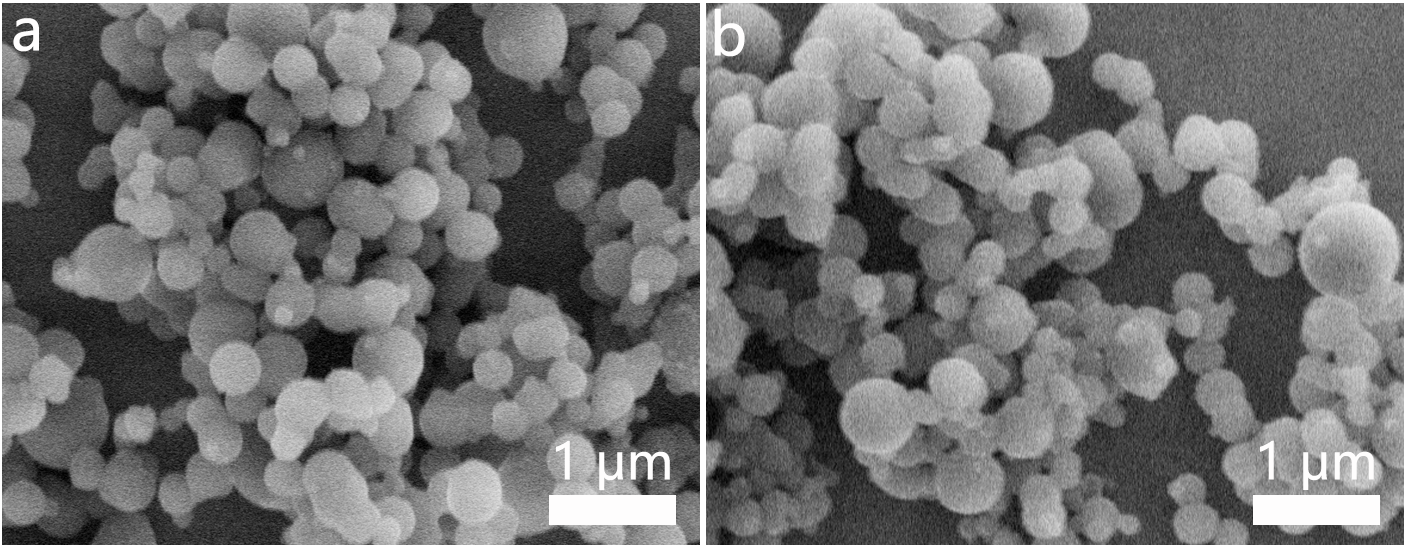


Figure S2. SEM images of the covalent self-assembled peptide TTP5-Random.


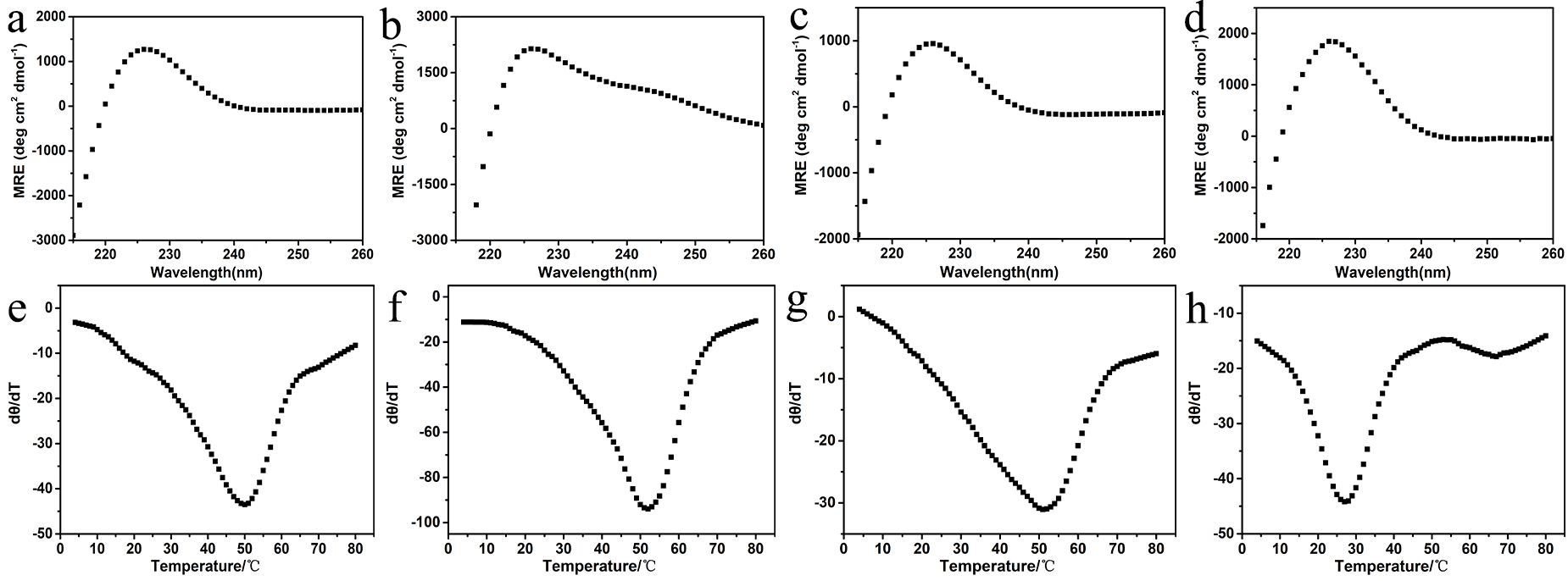


Figure S3. CD characterization of peptides TTP7 (a, e), TTP8 (b, f), TTP9 (c, g) and TTP10 (d, h). CD spectra (a-d) and the d[θ]/dT of their CD thermal unfolding curves (e-h).


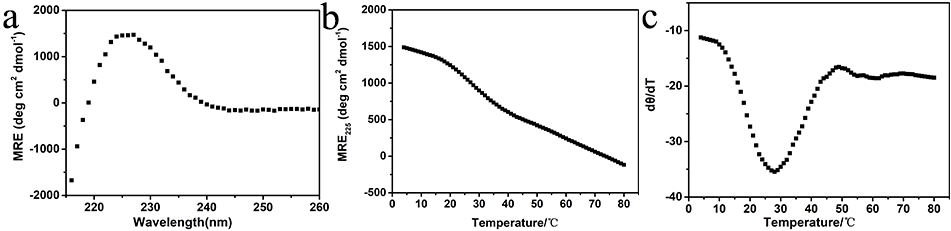


Figure S4. CD characterization of peptide TTP11. CD spectra (a), CD thermal unfolding curve (b) and the d[θ]/dT of the CD thermal unfolding curve (c).


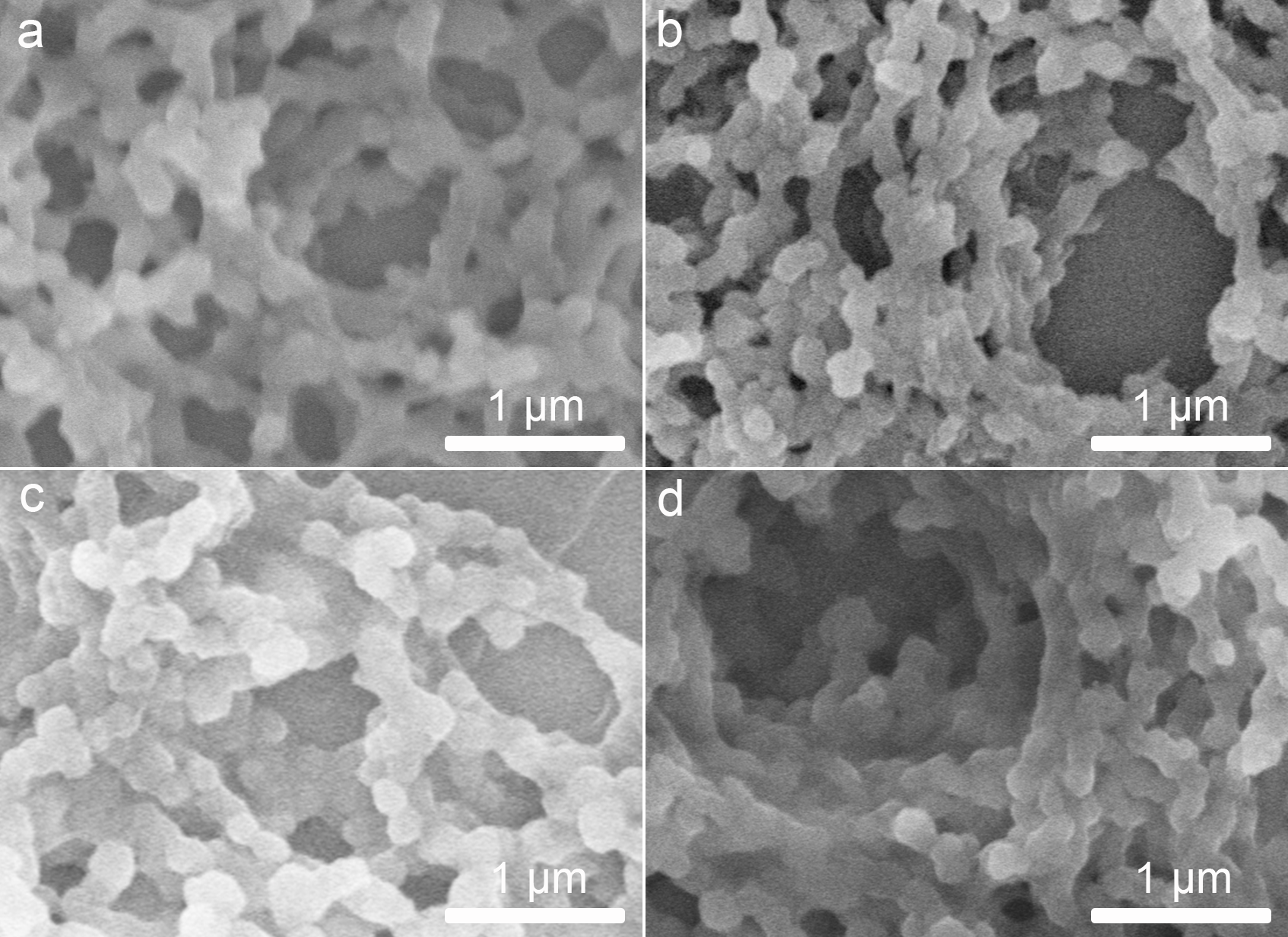


Figure S5. SEM images of TTP11 assemblies incubated at different pHs: pH 4.0 (a), pH 6.0 (b), pH 8.0 (c) and pH 10.0 (d) for 24 hrs at room temperature.


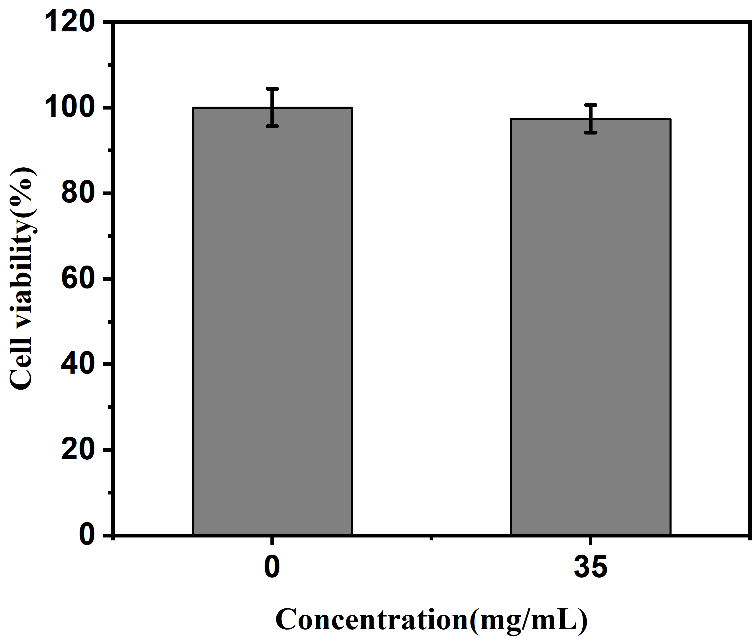


Figure S6. In vitro cytotoxicity of TTP11 assemblies at 35mg/mL.
